# Animal Bite Injuries to the Face: A Retrospective Evaluation of 111 Cases

**DOI:** 10.3390/jcm12216942

**Published:** 2023-11-06

**Authors:** Michael Maurer, Cornelius Schlipköter, Maximilian Gottsauner, Waltraud Waiss, Johannes K. Meier, Mathias Fiedler, Johannes G. Schuderer, Juergen Taxis, Torsten E. Reichert, Tobias Ettl

**Affiliations:** Department of Cranio- and Maxillofacial Surgery, Hospital of the University of Regensburg, Franz-Josef-Strauß-Allee 11, 93053 Regensburg, Germany

**Keywords:** animal bite, wound infection, facial trauma, maxillofacial surgery

## Abstract

The treatment of bite wounds to the face is discussed controversially in relation to surgery and antibiotics. The aim of this study is a retrospective evaluation of 111 cases of animal bite injuries to the face that presented to our unit of oral and maxillofacial surgery over a 13-year period. Children under 10 years of age were predominantly involved. A total of 94.5% of the assessed injuries were caused by dogs. Wound infections occurred in 8.1%. Lackmann type II was the most common type of injury (36.9%). The perioral area was affected most frequently (40.5%). Primary wound closure was carried out in 74.8% of the cases. In 91.9% of the cases, antibiotic prophylaxis was prescribed. The most often administered type of antibiotic was amoxicillin with clavulanic acid (62.1%). Patients without antibiotics showed an increased infection rate without significance. Wound infections occurred significantly more frequently in wounds to the cheeks (*p =* 0.003) and when local flap reconstruction was necessary (*p =* 0.048). Compared to the other surgical treatment options, primary closure showed the lowest infection rates (4.8%, *p =* 0.029). We recommend antibiotic prophylaxis using amoxicillin with clavulanic acid and wound drains for wounds of Lackmann class II or higher. Primary closure seems to be the treatment of choice whenever possible.

## 1. Introduction

Bite-related injuries are a frequent cause of presentations in emergency departments. About 30.000–50.000 bite wounds are reported in Germany per year, mainly caused by dogs and cats [1]. An estimated 8500 bite wounds per year are located on the face [2]. From 50 to 75% of these accidents happen to children [3]. The most common complication of dog bites is an infection secondary to wound contamination by both gram-positive and gram-negative microorganisms in the saliva [4].

The management of bite wounds is discussed controversially, and the risk of infections and the recommendations of anti-infective treatment are vague, ranging from cleansing the wound over antibiotic prophylaxis to antibiotic treatment in the case of wound infection [5]. Some studies do not deem routine antibiotic therapy in facial bite wounds as necessary [6]. Others report the use of broad-spectrum antibiotics in all cases of dog bites [7]. Concerning surgical treatments, previous studies suggest, for example, that primary closure of facial dog bites in children can be achieved with a low infection rate and an excellent cosmetic outcome [8]. Unfortunately, major reconstructive surgery is required in rare cases of animal attacks as a result of high masticatory forces and large breeds [9]. To improve the management of facial animal bite wounds, we retrospectively analysed the epidemiological aspects as well as the surgical and antibiotic treatment of the patient population presenting in our department of oral and maxillofacial surgery with bite wounds to the face.

## 2. Patients and Methods

From 2009 to 2022, 111 patients presented with bite injuries to the face in our Department of Oral and Maxillofacial Surgery at Regensburg University Hospital. Epidemiologic data were collected from patients’ medical records. Lackmann’s classification was used to define wound severity [10] (Table 1). The main wound locations were categorized as nasal, periorbital, perioral, cheek, auricular or frontotemporal area. Intraoral perforation, tissue defect and lacrimal duct involvement were assessed. Surgical treatment was categorised into solitary wound-cleansing, primary wound closure, local flap reconstruction, reconstruction with skin and cartilage grafts and microsurgical replantation and free flap reconstruction. Criteria for wound infection were fever (over 38 °C), lymphangitis, abscess or at least four of five minor criteria: erythema, tenderness, swelling at the wound-site, purulent secretion and leukocytosis of more than 12 × 10^9^/L [5,11]. Antibiotic therapy was assessed regarding the type of antibiotics and the treatment onset. A total of 100 patients were treated within 6 h of the trauma, 11 were treated after 6 h or more but not later than within the day after the trauma. If required, scar correction was performed at least 6 months after the primary surgical treatment. Comorbidities that affected the results could not be identified. Data were analysed by the use of SPSS 26.0 (IBM Corp., Armonk, NY, USA). Significant differences were identified in cross-tabulation using Pearson’s chi-square-test, correlations and the Mann–Whitney U Test. A *p*-value less than 0.05 was considered as statistically significant.

## 3. Results

### 3.1. Epidemiology

**Figure 1:**
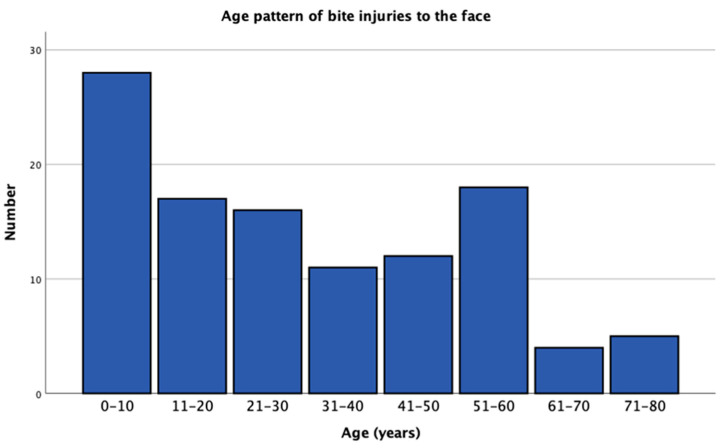
Age pattern of patients suffering animal bites to the face.

Table 2 shows the base data of the entire cohort. A total of 59 female and 52 male patients underwent treatment for animal bite injuries to the face from 2009 to 2022 in our unit. The mean age was 30.30 ± 21.50 years (range 1–76 years). A total of 28 patients were 10 years or less (25.2%) and represented the dominant age band (Figure 1). In 105 cases (94.5%), bites were caused by dogs, in 5 cases by horses (4.5%) and in one case by a fox (0.9%). Only female patients sustained horse bites (*p* = 0.032). A total of 64% of the involved animals (n = 71) were familiar to the victims. In 18.0% of the involved dogs, complete vaccination status including rabies was documented (n = 20), and in 82.0% of cases, the animal´s vaccination status was unknown (n = 91).

### 3.2. Wound Characteristics

**Figure 2:**
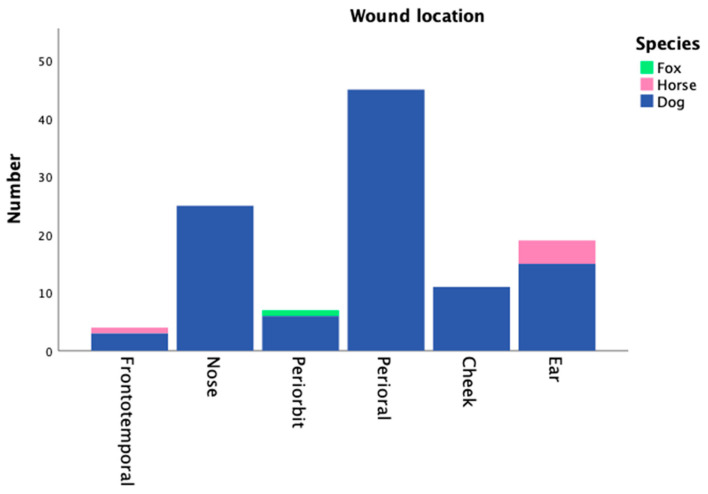
Wound locations.

Wound characteristics are shown in Table 3. The main location of the wounds was perioral (n = 45; 40.5%), followed by the nose (n = 25; 22.5%), ear (n = 19; 17.1%) and cheek (n = 11; 9.9%). Other locations were periorbital (n = 7; 6.3%) and frontotemporal (n = 4; 3.6%) (Figure 2). Horse bites were mainly located in the ear and the frontotemporal area, whereas all of the perioral wounds were caused by dogs. This distribution was significant (*p* < 0.001). The dominant type of injury pattern was Lackmann type II (n = 42; 37.8%), followed by type III (n = 37; 33.3%) and type I (n = 29; 26.1%). Lackmann type IV was present in 3 cases (IVa: n = 1; 0.9%, IVb: n = 2; 1.8%). Lackmann type was not associated with the presence of infections (*p =* 0.750). Perforation to the oral cavity was noted in 14 cases (12.6%). Defect wounds occurred in 37 cases (33.3%). In 6 cases, merely wound cleansing was required (5.4%).

### 3.3. Surgical Treatment

**Figure 3:**
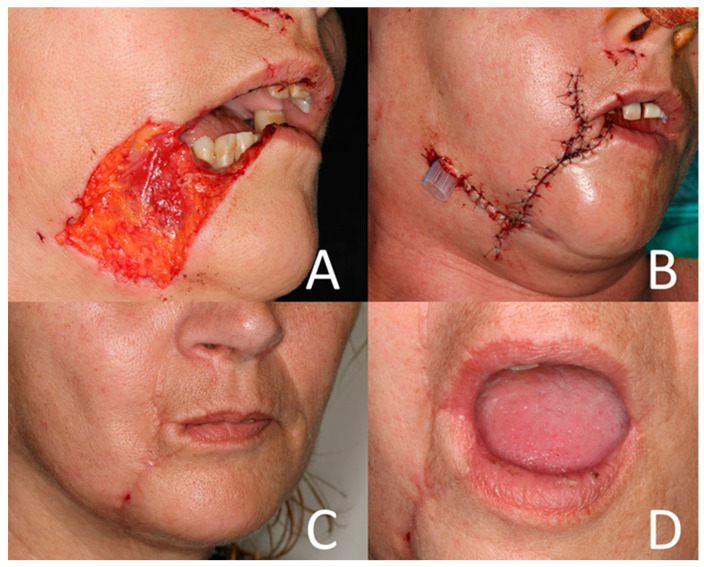
(**A**) Lackmann type III bite injury after a dog attack. (**B**) Local flap reconstruction. Drain installed at lateral wound site. (**C**) Situation 3 months after surgery. (**D**) Mouth opening 3 months after surgery.

In 83 cases, debridement and primary closure were carried out (74.8%). Local flap reconstruction was accomplished in 5 cases (4.5%) (Figure 3A–D), avascular grafts were required in 12 cases (10.8%) and microsurgical/free flap reconstruction in 5 cases (4.5%). In 42 cases, general anaesthesia for surgical treatment was necessary. Wound drains were placed in 21 cases (18.9%). In 95.2%, drains were placed in wounds classified as Lackmann type II or higher (n = 20) (Figure 3B). This finding was statistically significant (*p =* 0.013). A total of 8 patients underwent scar correction at least 6 months after primary trauma management (7.2%) (Table 4).

### 3.4. Antibiotic Treatment

Table 5 gives an overview of the antibiotic treatment of the entire cohort. A total of 102 patients received antibiotic prophylaxis (91.0%). A total of 9 patients did not obtain antibiotic prophylaxis (8.1%), and 2 of them received delayed antibiotic treatment because of wound infection. A total of 69 patients were prescribed amoxicillin with clavulanic acid (62.1%), 15 clindamycin (13.5%), 12 cefuroxim (10.8%), 4 cefazolin (3.6%), one penicillin (0.9%) and one ciprofloxacin (0.9%). In 3 cases, metronidazol was added to one of the first-mentioned antibiotic agents (2.7%).

### 3.5. Wound Infections

A total of 7 of the antibiotically treated patients developed a wound infection (6.8%). In the patient group without antibiotic prophylaxis, 2 of 9 patients showed signs of infection (22.2%). This difference was not statistically significant (*p =* 0.818). A higher infection rate in patients with delayed surgical treatment could also not be displayed (*p =* 0.90). Scar correction surgery was significantly required more often in patients with wound infection after the initial treatment (*p =* 0.002). Compared to the other surgical treatment options, direct wound closure led to significantly less need for scar corrections (2.4%; *p =* 0.001). In 14 cases, a perforation into the oral cavity was described (12.6%). One of these perforating wounds led to an infection (7.1%). There was no significant correlation between oral perforation and wound infection (*p =* 0.887). In 4 of the 37 defect wounds, signs of infection were recognized (10.8%) without achieving statistical significance (*p =* 0.461). In 3 of the 21 drained wounds, signs of infection were detected (14.3%) (*p =* 0.249). Regarding surgical treatment, local flap reconstruction led to the highest percentage of infection (2 of 5 cases, 40%). The distribution of infections dependent on the different ways of surgical treatment was significant (*p =* 0.048). By comparing primary closure to all other treatment options, a significantly lower rate of infection in the primarily closed wounds could be displayed (*p =* 0.029) (Table 6). Concerning the Lackmann stage, the highest infection rate was found in stage II wounds (11.9%), followed by stage III (8.1%). A significant association between the Lackmann stage and wound infection could not be ascertained (*p* = 0.750).

## 4. Discussion

Up to 30,000–50,000 injuries per year are associated with animal bites in Germany. A total of 60–80% of these injuries result from dog bite injuries [1]. Approximately one in twenty dogs will bite a human being during his or her lifetime [4]. Following the upper and lower extremities, the face is the most common area for bite injuries [12,13]. Especially in children, the face is reported to be the most common location of bite wounds [14]. Facial injury complications following animal bites include soft tissue infections and prominent scars [15]. In our own department, there were 111 patients with animal bite wounds to the face over the 13-year period documented. A total of 94.5% of the bite wounds were caused by dogs and 4.5% by horses. Interestingly, cat bites were not reported. They seem to be located more commonly on the hands followed by the upper and lower extremities [16]. Regarding the patients’ age pattern, the dominant group were children between 0 and 10 years (25.2%). Other authors also report children to be at the highest risk of falling victim to dog bites [4,5,17]. This is likely caused by the unintentionally threatening and provoking behaviour of children against dogs [3]. Another reason is probably the smaller size of children and their faces being in the range of medium- and large-sized dogs [18]. In this context, children are reported to be two times more likely to suffer a periorbital injury from dog attacks when compared to adults [19].

The most common site was the perioral region (40.5%), followed by the nose (22.5%) and the ear (17.1%). This is basically consistent with other studies [5,20,21] and may be caused by their exposed location. Horse bites mainly addressed the periauricular and frontotemporal area, whereas injuries especially in the perioral and nasal tissues were exclusively caused by dogs (*p* < 0.001). This might result from the different directions of the attacks. Dogs commonly attack from the bottom up, so the victim’s perioral tissue is more reachable for them. Horse bites were exclusively noticed in female patients (*p* = 0.032) as, in our region, the most common contact with horses originates from horseback riding.

An overall infection rate of 8.1% was detected. This is consistent with previous studies [22,23]. The significantly highest percentage of infections was registered in wounds affecting the cheeks compared to all other facial soft tissues (36.4%; *p* = 0.006). Guo et al. also identified soft tissue injuries to the cheek to be more at risk for infection compared to injuries to other facial areas, independent of the cause of the accident [24]. Stanbouly et al. identified the cheeks as the most frequent site to develop open wounds caused by dog bites and that open wounds are more likely to develop an infection following dog bites [18]. We suppose the complex multi-layer anatomy of the cheek to be responsible for that.

With regard to the different ways of surgical treatment, a slight increase in infections in patients undergoing local flap reconstruction could be detected (*p* = 0.048). Local flap reconstruction was exclusively required in stage II and III wounds. The larger defect size and the involvement of deep tissues may be responsible for the higher infection rate in these cases. To prevent infections, we recommend installing a drain in cases of local flap reconstruction. Primary closure seems to cause the lowest infection rate (4.8%, *p* = 0.029). Regarding aesthetic outcomes, scar correction was significantly less required after direct closure compared to the other surgical treatment options (*p* = 0.001). Coinciding with other authors, we recommend prompt primary wound closure after careful cleansing and disinfection if possible [23]. Detailed information about eventual complications such as wound infection and hypertrophic scarring has to be provided preoperatively to the patients to avoid future complaints [25]. Another interesting treatment option is described by Lisong et al., who recommend the application of medical glue after negative pressure sealing and drainage to treat children’s maxillofacial dog bites. The use of medical glue is time-saving, leads to smooth scars and high satisfaction, especially in children and their families, and should be integrated into clinical routine in the case of animal bite injuries to the face [22].

Despite previous authors’ reports of higher infection rates in intra-oral/extra-oral communicating wounds and because of the additional exposure to the victim’s own oral flora [9,24], in the present study oral perforation was not a promoting factor for infection. Nevertheless, in these cases, we advise proper wound cleaning from extraoral and intraoral and watertight closure of the intraoral aspect of the wound to prevent additive contamination by their own salivary flora.

Regarding the need for antibiotic treatment, Kesting et al. recommend antibiotic prophylaxis for all wounds of Lackmann class II or higher, in cat and horse bites, in children, in patients with immunodeficiency and in wounds older than 6 h [5]. Others advise the early prescribing of prophylactic oral antibiotics in all cases of bite injuries [26,27,28]. In our department, antibiotic prophylaxis was administered to 91.9% of patients presenting with bite injuries to the face (n = 102). A total of 6.9% of them showed signs of wound infection despite prophylactic antibiotic treatment (n = 7). Nine patients did not receive an antibiotic prophylaxis and two of them developed a wound infection (22.2%). This difference was not significant (*p* = 0.197). The patients’ age did not seem to influence the development of wound infections as children under 10 years nearly showed the same infection rates (8.0%) as patients older than 10 years (8.1%). Regarding wound infection according to the Lackmann classification, the lowest infection rate was assessed in class I wounds (3.4%), whereas the most infections were documented in class II (11.9%) and III (8.1%). A significant correlation between the Lackmann stage and wound infection could not be displayed (*p* = 0.750). In stage I wounds, only one infection in 29 wounds was detected, whereas in stages II and III the percentage of infections obviously increased. In stage IV wounds, no infections were assessed. However, this finding can be attributed to the low number of stage IV wounds (n = 3) and may not be representative. With this in mind, our findings indicate that the risk for wound infection in stage I wounds is low and increases with the involvement of deep tissues. According to these results, we accede to the proposal of Kesting et al. for antibiotic prophylaxis for patients with Lackmann class II or higher facial bite injuries. In contrast, proper local disinfection seems to be appropriate in Lackmann class I cases after careful evaluation of the individual situation concerning the patient’s immunological competence, macroscopic wound contamination, etc. We would not suggest a special need for preventive antibiotic use in children as infection rates in children seem to be equal to adults. In other studies, the evaluation of the complications revealed that hypertrophic scarring was the most common complication following surgery [21]. A total of eight patients required dermal scar correction after at least 6 months (7.2%). The percentage of patients undergoing scar correction was significantly increased in patients with wound infection documented compared to patients with complication-free wound healing (*p* = 0.001). We suppose that wound infections lead to enhanced scarring and reduced long-term aesthetics. In 21 cases, a wound drain was inserted as a part of wound closure or reconstruction surgery. A total of 14.2% of the wounds with a drain showed signs of infection (n = 3 of 21) compared to the 6.7% infection rate in wounds without a drain (n = 6 of 90). However, there was a significant correlation between Lackmann class II or higher and the installation of a drain (*p* = 0.013). So, the higher infection rate could be explained by the fact that drains were mainly installed in critical wounds with a higher risk of infection. So, despite this higher occurrence of infections, we recommend inserting a drain in visibly contaminated wounds and optionally in Lackmann class II or higher.

Common pathogens associated with animal bites include *Staphylococcus*, *Streptococcus*, *Pasteurella*, *Capnocytophaga*, *Moraxella*, *Corynebacterium*, *Neisseria* and *Anaerobic* bacteria [11]. Dog bites can result in the transmission of numerous pathogens including *Rabies lyssavirus* (i.e., rabies), *Clostridium tetani* (i.e., tetanus), *Pasteurella* spp., *Capnocytophaga canimorsus*, *Fusobacterium*, *Bacteroides*, *Prevotella* spp., *Propionibacterium*, *Peptostreptococcus*, *Eikenella corrodens* and *Streptococcus pyogenes*, among others [18,29]. Amoxicillin with clavulanic acid is generally considered the first-line prophylactic treatment for animal bites [21,30,31]. Amoxicillin is a penicillin derivative and has a similar activity against both gram-positive and gram-negative bacteria. With the addition of clavulanic acid, the spectrum is increased to include beta-lactamase-producing strains as well as broadening the coverage to include other bacterial species [32]. According to this, the most frequently administered antibiotic agent was amoxicillin with clavulanic acid (n = 69). Amoxicillin with clavulanic acid is reported to be virtually active against all the bacteria isolated from bite wounds [5,33]. When given with a prophylactic intention, wound infection occurred in 7.2% (n = 5). This low number of infections supports amoxicillin with clavulanic acid and seems to be the agent of choice as the first option in all facial bite injuries. Prophylactic antibiotics should be prescribed for 3 days [34]. If a wound shows evidence of infection, a microbiology swab should be taken for culture and sensitivity [34]. Antibiotics for treatment of infection should be prescribed for 5 days [34]. As alternatives to amoxicillin with clavulanic acid, mainly clindamycin and cefuroxim were administered. Clindamycin is well known for its activity against anaerobic bacteria, particularly beta-lactamase-producing strains of the Bacteroides species and its activity against aerobic gram-positive cocci. However, clinicians should be aware of its failure against aerobic gram-negative rods [35]. Cefuroxime is stable to many β-lactamases and is active against many gram-positive and gram-negative organisms. Like most other cephalosporins, it is not active against *Streptococcus faecalis*, *Pseudomonas* species or *Bacteroides* species [36]. Tetanus and rabies immunization history must be checked, and vaccination and immune globulin should be administered when necessary. According to the recommendations of the WHO, nibbling of uncovered skin, minor scratches or abrasions without bleeding and licks on slightly abraded skin demand immediate post-exposition vaccination and local treatment of the wound. Single or multiple transdermal bites or scratches (with bleeding), licks on broken skin, contamination of mucous membrane with saliva from licks and contact with bats (superficial or deep bites or scratches, contact with a wound or mucous membrane) require immediate post-exposure vaccination and the administration of immunoglobulins [37]. In the present cohort, rabies immunization was carried out in the case of a fox bite. Since 2008, Germany has been considered to be free from terrestrial rabies. Nevertheless, post-exposure prophylaxis should be carried out if the suspicion of being exposed to rabies cannot be invalidated as it was in our case [38]. It must be remembered that, in other countries, emergency physicians have to cope with a more difficult situation concerning rabies related to a high number of straining dogs. For instance, Aydin et al. report in a Turkish study that 97.1% of patients presenting with bite injuries receive a rabies vaccination [13].

Another interesting aspect of this study is the fact that long-term facial nerve malfunctions after suffering a bite injury were not explicitly recorded. The incidence of permanent facial nerve harm after animal attacks to the face seems to be quite low. However, further research is required for a detailed assessment of the function of the facial nerve in patients with facial bite injuries.

A limitation of the study is the retrospective design over a 13-year time period. Despite accurate documentation detailed information about initial medical findings, treatments and outcomes may be absent. Several additional cases had to be excluded because of incomplete medical records. In this context, data about patients’ comorbidity and smoking status are missing. This means a major compromising factor to the treatment outcomes and the results of the study. Regarding the surgical treatment, information about the use of specific disinfection agents could not be achieved. Therefore, it could not be displayed which disinfection concept is appropriate for animal bite wounds. Moreover, treatment was carried out by multiple practitioners. Individual surgical experience might affect treatment outcomes but could not be reflected in the study’s results. Another limitation of the study is an incomplete microbiological assessment, as the cultivation of bacteria causing wound infections was successful just in three cases. Cultures of bite wounds are not obligate initially, unless the wound is abscessed or already infected [39]. In our cohort, microbiological cultures were not collected routinely from wounds not infected. The lack of bacteria cultures from infected wounds may be promoted by the reflexive empirical administration of broad-spectrum antibiotics before taking swab specimens of the wounds.

## 5. Conclusions

Animal bite wounds to the face are a common reason for presentation in emergency departments. Children under 10 years of age are the main portion of the patient population. The main location is the perioral region, followed by the nose and ear. Cheek wounds are at greatest risk for wound infection as well as local flap reconstruction. Perforation wounds into the oral cavity do not imply increased infection rates. We recommend antibiotic prophylaxis with amoxicillin with clavulanic acid and wound drains for wounds of Lackmann class II or higher. Primary closure of the wounds seems to be the treatment of choice if possible concerning infection rates and aesthetic outcomes.

## Figures and Tables

**Table 1:** Lackmann’s classification of bite wounds.

Stage	Clinical Features
I	Superficial injury not involving muscle
II	Deep injury involving muscle
III	Deep injury involving muscle with loss of tissue
IVa	Deep injury involving muscle with loss of tissue and injury to vessels or nerves
IVb	The above, and bone involvement

**Table 2:** Base data.

**Total**	111
**Mean Age**	30.30 ± 21.50 years
**Sex**	
Female	59 (53.2%)
Male	52 (46.8%)
**Species**	
Dog	105 (94.6%)
Horse	5 (4.5%)
Fox	1 (0.9%)
**Animal familiar**	
Yes	71 (63.9%)
No	40 (36.0%)
**Treatment delay**	
Treatment within 6 h	100 (90.1%)
Treatment after 6 h or more	11 (9.9%)
**Immunisation status animal**	
Complete	20 (18.0%)
Unknown	91 (82.0%)

**Table 3:** Wound characteristics.

**Main Location Wound**	
Frontotemporal	4 (3.6%)
Nose	25 (22.5%)
Periorbit	7 (6.3%)
Perioral	45 (40.5%)
Cheek	11 (9.9%)
Ear	19 (17.1%)
**Oral perforation**	
Yes	14 (12.6%)
No	97 (87.4%)
**Lackmann classification**	
I	30 (27.0%)
II	41 (36.9%)
III	37 (33.3%)
IVa	1 (0.9%)
IVb	2 (1.8%)
**Tissue defect**	
Yes	40 (36.0%)
No	71 (64.0%)
**Fracture**	
Yes	2 (1.8%)
No	109 (98.2%)

**Table 4:** Surgical treatment.

**Anaesthesia**	
Local	69 (62.2%)
General	42 (37.8%)
**Surgical treatment**	
Wound-cleansing alone	6 (5.4%)
Debridement and primary closure	83 (74.8%)
Local flap reconstruction	5 (4.5%)
Avascular graft reconstruction	12 (10.8%)
Microsurgical replantation or free flap reconstruction	5 (4.5%)
**Wound drain**	
Yes	21 (18.9%)
No	90 (81.1%)
**Lacrimal duct reconstruction**	
Yes	2 (1.8%)
No	109 (98.2%)
**Scar correction required**	
Required	8 (7.2%)
Not required	103 (92.8%)
After primary closure	2/83 (2.4%)
After other surgical treatment	6/28 (21.4%)

**Table 5:** Antibiotic treatment.

**Antibiotic Prophylaxis**	
No	9 (8.1%)
Yes	102 (91.9%)
**Type of antibiotics**	
Amoxycillin with clavulanic acid	69 (62.1%)
Clindaymcin	15 (13.5%)
Cefuroxim	12 (10.8%)
Cefazolin	4 (3.6%)
Penicilline	1 (0.9%)
Ciprofloxacin	1 (0.9%)
Metronidazol (additional)	3 (2.7%)

**Table 6:** Wound infections.

**Wound Infection**		
Yes	9 (8.1%)	
No	102 (91.9%)	
**Cases of infection/Wound location**		*p* = 0.003
Nose	1/25 (4.0%)	
Periorbit	1/7 (14.3%)	
Perioral	1/45 (2.3%)	
Cheek	4/11 (36.4%)	
Ear	1/19 (5.2%)	
Frontotemporal	1/4 (25%)	
**Scar correction required**		*p* = 0.001
After wound infection	3/9 (33.3%)	
Without wound infection	5/102 (4.9%)	
**Infections in**		*p* = 0.887
Wounds with oral perforation	1/14 (7.1%)	
Wounds without oral perforation	8/97 (8.2%)	
**Infections in**		*p* = 0.461
Defect wounds	4/37 (10.8%)	
Wounds without tissue defect	5/74 (6.8%)	
**Infections in**		*p* = 0.212
Wounds with drain	3/20 (15.0%)	
Wounds without drain	6/91 (6.6%)	
**Infections in**		*p* = 0.900
Wounds treated within 6 h	8/100 (8.0%)	
Wounds treated after 6 h	1/11 (9.1%)	
**Infections in wounds treated by**		*p* = 0.048
Cleansing alone	1/6 (16.7%)	
Primary closure	4/83 (5.1%)	
Local flap reconstruction	2/5 (40%)	
Reconstruction with avascular graft	1/12 (8.3%)	
Microsurgical replantation or free flap reconstruction	1/5 (20%)	
**Infections in wounds treated by**		*p* = 0.029
Primary closure	4/83 (4.8%)	
Other treatment	5/28 (17.9%)	
**Infections/Lackmann Classification**		*p* = 0.750
I	1/29 (3.4%)	
II	5/42 (11.9%)	
III	3/37 (8.1%)	
IVa	0/1 (0%)	
IVb	0/2 (0%)	
**Infections in patients with**		*p* = 0.197
antibiotic prophylaxis	7/104 (6.7%)	
without antibiotic prophylaxis	2/7(28.6%)	
**Infection in patients**		*p* = 0.982
age under 10 years	2/25 (8.0%)	
age 10 or older	7/86 (8.1%)	
**Infection/Antibiotic prophylaxis**		*p* = 0.407
None	2/9 (22.2%)	
Amoxycillin with clavulanic acid	5/69 (7.2%)	
Clindaymcin	0/15 (0%)	
Cefuroxim	1/12 (0%)	
Cefazolin	1/4 (25%)	
Penicilline	0/1 (0%)	
Ciprofloxacin	0/1 (0%)	
Metronidazol (additional)	0/3 (0%)	

## Data Availability

Data can be obtained on request by the scientists who conducted the work independently from the industry. Data are not stored on publicly available servers.

## References

[1] Rothe K., Tsokos M., Handrick W. (2015). Animal and Human Bite Wounds. Dtsch. Arztebl. Int..

[2] Herbert I., Bünger B. (1986). Hundebissverletzungen im Kopf-Hals-Bereich. Laryngol. Rhinol. Otol..

[3] Boenning D.A., Fleisher G.R., Campos J.M. (1983). Dog bites in children: Epidemiology, microbiology, and penicillin prophylactic therapy. Am. J. Emerg. Med..

[4] Griego R.D., Rosen T., Orengo I.F., Wolf J.E. (1995). Dog, cat, and human bites: A review. J. Am. Acad. Dermatol..

[5] Kesting M.R., Hölzle F., Pox C., Thurmüller P., Wolff K.-D. (2006). Animal bite injuries to the head: 132 cases. Br. J. Oral Maxillofac. Surg..

[6] Wolff K.D. (1998). Management of animal bite injuries of the face: Experience with 94 patients. J. Oral Maxillofac. Surg..

[7] Méndez Gallart R., Gómez Tellado M., Somoza Argibay I., Liras Muñoz J., Pais Piñeiro E., Vela Nieto D. (2002). Mordeduras de perro. Análisis de 654 casos en 10 años. An. Esp. Pediatr..

[8] Wu P.S., Beres A., Tashjian D.B., Moriarty K.P. (2011). Primary repair of facial dog bite injuries in children. Pediatr. Emerg. Care.

[9] Stefanopoulos P.K., Tarantzopoulou A.D. (2005). Facial bite wounds: Management update. Int. J. Oral Maxillofac. Surg..

[10] Lackmann G.M., Draf W., Isselstein G., Töllner U. (1992). Surgical treatment of facial dog bite injuries in children. J. Craniomaxillofac. Surg..

[11] Talan D.A., Citron D.M., Abrahamian F.M., Moran G.J., Goldstein E.J. (1999). Bacteriologic analysis of infected dog and cat bites. Emergency Medicine Animal Bite Infection Study Group. N. Engl. J. Med..

[12] Ferreira S., Ayres Quaresma L.E., Timóteo C.A., da Silva Fabris A.L., Faverani L.P., Francisconi G.B., Souza F.A., Júnior I.R.G. (2014). The primary closure approach of dog bite injuries of the nose. J. Craniofac. Surg..

[13] Aydin O., Aydin Goker E.T., Arslan Z.A., Sert H.M., Teksam O. (2023). Clinical features and management of animal bites in an emergency department: A single-center experience. Postgrad. Med..

[14] Mattice T., Schnaith A., Ortega H.W., Segura B., Kaila R., Amoni I., Shanley R., Louie J.P. (2023). A Pediatric Level III Trauma Center Experience with Dog Bite Injuries. Clin. Pediatr..

[15] Becerra C.M.C., Hodge D.O., Bradley E.A. (2023). Incidence and Characteristics of Facial and Ophthalmic Injuries from Domestic Mammal Bites: Parts of the data in the manuscript were presented at the American Academy of Ophthalmology Annual Meeting, 2022. Am. J. Ophthalmol..

[16] Palacio J., León-Artozqui M., Pastor-Villalba E., Carrera-Martín F., García-Belenguer S. (2007). Incidence of and risk factors for cat bites: A first step in prevention and treatment of feline aggression. J. Feline Med. Surg..

[17] Selvi F., Stanbouly D., Stanbouly R., Baron M., Francois K., Halsey J., Marx R.E., Chuang S.-K. (2022). Early Childhood (0 to 5 years) Presents the Greatest Risk for Facial Dog Bites. J. Oral Maxillofac. Surg..

[18] Stanbouly D., Stewart S.J., Harris J.A., Chuang S.-K. (2023). Risk factors associated with infection in patients sustaining dog bites to the face. Oral Maxillofac. Surg..

[19] Hwang M., Engelstad M., Chandra S.R. (2023). Management of Soft Tissue Injuries in Children—A Comprehensive Review. Oral Maxillofac. Surg. Clin. N. Am..

[20] Ali S.S., Ali S.S. (2022). Dog bite injuries to the face: A narrative review of the literature. World J. Otorhinolaryngol. Head Neck Surg..

[21] Chen T., Karim M., Grace Z.T., Magdich A.R., Carniol E.C., Benson B.E., Svider P.F. (2023). Surgical management of facial dog bite trauma: A contemporary perspective and review. World J. Otorhinolaryngol. Head Neck Surg..

[22] Lisong H., Lianfu W., Jinhong Y., Haibin Z. (2023). Clinical effect analysis of using medical glue versus conventional suturing to treat dog bite in children’s maxillofacial region after negative pressure sealing drainage: A randomized trial. Medicine.

[23] Paschos N.K., Makris E.A., Gantsos A., Georgoulis A.D. (2014). Primary closure versus non-closure of dog bite wounds. A randomised controlled trial. Injury.

[24] Guo H.-Q., Yang X., Wang X.-T., Ji A.-P., Bai J. (2022). Risk Factors for Infection of Sutured Maxillofacial Soft Tissue Injuries. Surg. Infect..

[25] Bracaglia R., D’Ettorre M., Gentileschi S., Tambasco D. (2014). Was the surgeon a satisfactory informant? How to minimize room for claims. Aesthet. Surg. J..

[26] Tabaka M.E., Quinn J.V., Kohn M.A., Polevoi S.K. (2015). Predictors of infection from dog bite wounds: Which patients may benefit from prophylactic antibiotics?. Emerg. Med. J..

[27] Monroy A., Behar P., Nagy M., Poje C., Pizzuto M., Brodsky L. (2009). Head and neck dog bites in children. Otolaryngol. Head Neck Surg..

[28] Cummings P. (1994). Antibiotics to prevent infection in patients with dog bite wounds: A meta-analysis of randomized trials. Ann. Emerg. Med..

[29] Greene C.E. (2023). Greene’s Infectious Diseases of the Dog and Cat.

[30] Morgan M., Palmer J. (2007). Dog bites. BMJ.

[31] Stevens D.L., Bisno A.L., Chambers H.F., Everett E.D., Dellinger P., Goldstein E.J.C., Gorbach S.L., Hirschmann J.V., Kaplan E.L., Montoya J.G. (2005). Practice guidelines for the diagnosis and management of skin and soft-tissue infections. Clin. Infect. Dis..

[32] Evans J., Hanoodi M., Wittler M. (2023). StatPearls: Amoxicillin Clavulanate.

[33] Goldstein E.J. (1999). Current concepts on animal bites: Bacteriology and therapy. Curr. Clin. Top. Infect. Dis..

[34] Fielding P., Messahel S. (2022). Guideline review—Human and animal bites: Antimicrobial prescribing. Arch. Dis. Child. Educ. Pract. Ed..

[35] Soper D.E. (1992). Clindamycin. Obstet. Gynecol. Clin. N. Am..

[36] (1989). Cefuroxime axetil: A new oral cephalosporin. DTB.

[37] WHO (2018). Rabies vaccines: WHO position paper, April 2018—Recommendations. Vaccine.

[38] Robert Koch-Insitut (2022). RKI Ratgeber Tollwut. Epid Bull..

[39] Ellis R., Ellis C. (2014). Dog and cat bites. Am. Fam. Physician.

